# Identification of Maize Genes Associated with Host Plant Resistance or Susceptibility to *Aspergillus flavus* Infection and Aflatoxin Accumulation

**DOI:** 10.1371/journal.pone.0036892

**Published:** 2012-05-14

**Authors:** Rowena Y. Kelley, W. Paul Williams, J. Erik Mylroie, Deborah L. Boykin, Jonathan W. Harper, Gary L. Windham, Arunkanth Ankala, Xueyan Shan

**Affiliations:** 1 Department of Biochemistry, Molecular Biology, Entomology, and Plant Pathology, Mississippi State University, Mississippi State, Mississippi, United States of America; 2 Corn Host Plant Resistance Research Unit, Agricultural Research Service, United States Department of Agriculture, Mississippi State, Mississippi, United States of America; 3 Statistics Office, Agricultural Research Service, United States Department of Agriculture, Stoneville, Mississippi, United States of America; 4 Department of Computer Science and Engineering, Mississippi State University, Mississippi State, Mississippi, United States of America; CNR, Italy

## Abstract

**Background:**

*Aspergillus flavus* infection and aflatoxin contamination of maize pose negative impacts in agriculture and health. Commercial maize hybrids are generally susceptible to this fungus. Significant levels of host plant resistance have been observed in certain maize inbred lines. This study was conducted to identify maize genes associated with host plant resistance or susceptibility to *A. flavus* infection and aflatoxin accumulation.

**Results:**

Genome wide gene expression levels with or without *A. flavus* inoculation were compared in two resistant maize inbred lines (Mp313E and Mp04∶86) in contrast to two susceptible maize inbred lines (Va35 and B73) by microarray analysis. Principal component analysis (PCA) was used to find genes contributing to the larger variances associated with the resistant or susceptible maize inbred lines. The significance levels of gene expression were determined by using SAS and LIMMA programs. Fifty candidate genes were selected and further investigated by quantitative RT-PCR (qRT-PCR) in a time-course study on Mp313E and Va35. Sixteen of the candidate genes were found to be highly expressed in Mp313E and fifteen in Va35. Out of the 31 highly expressed genes, eight were mapped to seven previously identified quantitative trait locus (QTL) regions. A gene encoding glycine-rich RNA binding protein 2 was found to be associated with the host hypersensitivity and susceptibility in Va35. A nuclear pore complex protein YUP85-like gene was found to be involved in the host resistance in Mp313E.

**Conclusion:**

Maize genes associated with host plant resistance or susceptibility were identified by a combination of microarray analysis, qRT-PCR analysis, and QTL mapping methods. Our findings suggest that multiple mechanisms are involved in maize host plant defense systems in response to *Aspergillus flavus* infection and aflatoxin accumulation. These findings will be important in identification of DNA markers for breeding maize lines resistant to aflatoxin accumulation.

## Introduction

The pathogenic fungus *Aspergillus flavus* draws considerable attention in agriculture because it produces aflatoxins that contaminate maize and other oilseed crops. Consumption of aflatoxin contaminated crops has been linked to liver cancer in animals and humans [1]. The U. S. Food and Drug Administration has set strict standards and regulations to control interstate commerce of aflatoxin contaminated feed and other products [2]. Aflatoxins are a group of polyketide-derived mycotoxins produced by toxigenic isolates of *Aspergillus flavus* and some other *Aspergillus* species upon colonization of host plants [3]. The fungal pathogenicity and the interactions between *Aspergillus flavus* and the host plant defense systems have been extensively studied in an effort to improve plant resistance and to reduce aflatoxin contamination [3], [4].

Plants respond to fungal pathogens through various defense mechanisms. The fungal pathogens are basically classified into groups of biotrophs, necrotrophs and hemibiotrophs [5]. The well studied gene-for-gene resistance system was usually observed in the interactions between biotrophic fungal pathogens and their host plants [5]. Upon such fungal infection, host plant resistance (R) proteins recognize the race-specific fungal elicitors and hence trigger a cascade of salicylate-dependent signal transduction pathways. As a result, the resistance reaction called hypersensitive response (HR) (localized lesion) takes place to limit fungal growth [6]–[8]. In contrast to the biotrophs, much less is known about the plant defense mechanism towards the necrotrophs. The plant resistance to necrotrophic fungal pathogens is likely controlled by quantitative resistance genes. Jasmonate- and ethylene-dependent signaling pathways are likely involved in such resistance systems. And they are triggered by fungal toxins or other fungal effectors [5]. Few resistance genes have been identified, and no gene-for-gene resistance systems have been reported in the necrotrophic fungal plant interactions [5]. Moreover, studies showed that the HR reactions actually enhance the host plant susceptibility and facilitate the necrotrophic fungal colonization [9]. Recently, a few structurally resistance-like (R-like) genes have been characterized to recognize fungal toxins and confer plant susceptibility during the necrotrophic fungal colonization (gene-for-gene susceptibility) [10], [11]. In such a case, host plant resistance exhibits in the form of insensitivity to fungal effectors in the plants that carry the mutant forms of the susceptibility-related R-like genes.

The toxigenic isolates of *Aspergillus flavus* possess the characteristics of necrotrophic fungal pathogens. Commercial maize hybrids are generally susceptible to *Aspergillus flavus*. The maize host resistance to *Aspergillus flavus* infection and aflatoxin accumulation has been identified in certain maize germplasm lines [12], [13]. Kernels of resistant maize lines show significantly less aflatoxin accumulation than susceptible maize lines at maturity. Previous studies on mapping populations derived from crosses between a resistant maize inbred line and a susceptible maize inbred line have led to the identification of several resistance-related quantitative trait loci (QTLs) in maize [14]–[16]. Most interestingly, QTLs for resistance to aflatoxin accumulation appeared to be derived from both resistant and susceptible parental lines (Willcox et al. 2000, unpublished data). Nevertheless, the molecular mechanisms underlying the significant aflatoxin reduction in resistant maize lines or the significant aflatoxin accumulation in susceptible maize lines are yet to be determined. To characterize maize genes involved in such host plant responses under *Aspergillus flavus* infection, we conducted a microarray analysis on kernel samples collected from a 4×2 factorial field experiment with two resistant maize inbred lines (Mp313E, Mp04∶86) in contrast to two susceptible inbred lines (Va35, B73). Candidate genes selected from the microarray analysis were further investigated by quantitative RT-PCR analysis between one resistant maize line (Mp313E) and one susceptible maize line (Va35) in a time course experiment.

## Results

### Statistical Analysis Revealed Gene Expression Patterns Specific to Resistant or Susceptible Maize Inbred Lines

Four maize inbred lines were used in this experiment. The field experimental design for all the maize inbred lines was a randomized complete block with split plot and three replications for each genotype. Table 1 shows comparison of the mean aflatoxin accumulation levels measured in mature maize kernels from the inoculated and un-inoculated primary ears of each genotype. Mp313E is a maize inbred line showing stable and inheritable resistance to *Aspergillus flavus* infection and low amount of aflatoxin accumulation in the kernels (Table 1). Va35 is a maize inbred line with good agronomic traits, but it shows susceptibility to *Aspergillus flavus* and high amounts of aflatoxin accumulation (Table 1). Mp04∶86 is a recombinant inbred line derived from a cross of maize lines Va35 (susceptible) and Mp715 (resistant). Thus Mp04∶86 shares genes from Va35, but it was selected for the trait of resistance to *Aspergillus flavus* and aflatoxin accumulation. B73 is an elite maize inbred line and a model resource for maize genome sequence information [17]. B73 is susceptible to *Aspergillus flavus* and also shows high amounts of aflatoxin accumulation (Table 1).

**Table 1:** Kernel aflatoxin levels in the four maize inbred lines used for the DNA microarray analysis.

Pedigree	Host Plant Response	Treatment	Aflatoxin(ng/g)
Mp04∶86	Resistant	Inoculated	195
		Uninoculated	∼ 1
Va35	Susceptible	Inoculated	1243
		Uninoculated	∼ 1
Mp313E	Resistant	Inoculated	140
		Uninoculated	∼ 1
B73	Susceptible	Inoculated	3791
		Uninoculated	∼ 1

**Figure 1:**
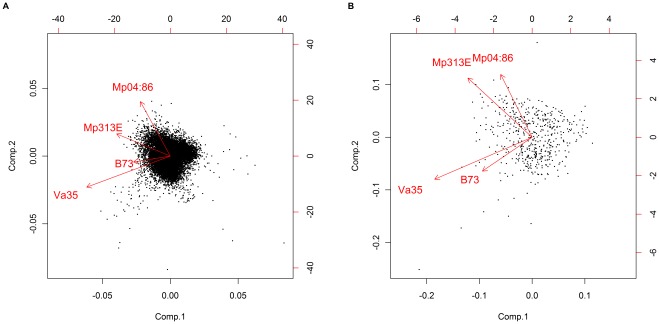
Biplots showing results of the principal component analysis (PCA) on log2 expression ratios (I/U) of 13,107 expressed genes. The distribution of the gene expression values shows evident trends represented by the vectors associated with resistant and susceptible maize inbred lines. Genes expressed toward the resistance trait were located close to the vectors for Mp313E and Mp04∶86. Genes contributing to susceptibility were located close to the vectors for Va35 and B73. 1A. Biplot of principal component analysis (PCA) on 13,107 expressed gene probes. 1B. Biplot of principal component analysis (PCA) on a subset of 500 expressed gene probes. (The arrows represent vectors. The direction and length represent the larger variance of the expression values).

The maize oligonucleotide arrays (from NSF Maize Oligonucleotide Array Project) used in this experiment contained 57,452 maize gene probes. In our study, each microarray slide was hybridized with two samples (dual channel hybridization) that were the inoculated and uninoculated samples from the same genotype and the same plot. In such an array design we first evaluated the resistance and susceptibility responses in each genotype and then compared the differentially expressed genes between groups of two resistant and two susceptible maize inbred lines. We performed multiple analyses on the microarray data using different statistical algorithms to reveal the host plant specific responses to *Aspergillus flavus* infection and aflatoxin reduction. We first analyzed microarray data with SAS Version 9.1.3 [18] using the mixed model for a split plot design with four maize inbred lines as main unit and two treatments [inoculated (I) vs. uninoculated (U)] as subunit. Log transformed median expression values were used to obtain estimates of gene expression ratios (I/U) for each genotype. Then the log2 values of the expression ratios (I/U) of 13,107 expressed genes from all genotypes were analyzed by principal component analysis (PCA). Figure 1A and 1B are biplots showing the distribution of the log2 gene expression ratios (I/U) on a projected principal plane. The data appeared to be cloudy, however, we found that the vectors representing the larger variances associated with the two resistant maize inbred lines (Mp313E and Mp04∶86) were clustered together. Likewise, the vectors representing the larger variances associated with the two susceptible maize inbred lines (Va35 and B73) were clustered together. That means despite the differences in the genomes of these genotypes, trends of gene expression associated with host plant resistance or susceptibility were evident. For example, Mp04∶86 showed a distinguished expression pattern compared to its susceptible parent Va35 in response to the fungal infection (Figure 1A and 1B). The PCA analysis has revealed a separation of genes between the resistant and susceptible groups in response to *Aspergillus flavus* colonization. Genes that expressed toward the larger variances were considered as associated with the corresponding traits. Genes expressed toward the resistance trait located close to the vectors for Mp313E and Mp04∶86. Genes for susceptibility located close to the vectors for Va35 and B73 (Figure 1A and 1B). By this method, we grouped genes for possible candidates contributing to either the host resistance or the host susceptibility.

As a computational validation step and an exploratory method to look at effects of different algorithms on test of the significance levels, we analyzed the microarray data using the R package of the Linear Models for Microarray Data (LIMMA) [19]. LIMMA uses Bayes method which is a different algorithm for identification of significantly expressed genes. Both SAS and LIMMA methods yielded large number of differentially expressed genes. To screen for the genes that have larger impacts on the traits and to reduce the number of less relevant genes, the lists of top ranked genes from LIMMA analysis were compared with the lists obtained from the analysis with SAS. Genes significant (P<0.01) from the LIMMA lists and ranking high on the SAS lists were selected. Final candidate genes for further quantitative analysis with qRT-PCR were selected by integration of the results from PCA, LIMMA and SAS analysis.

### Time Course Quantitative Expression Analysis on Selected Candidate Genes

**Figure 2:**
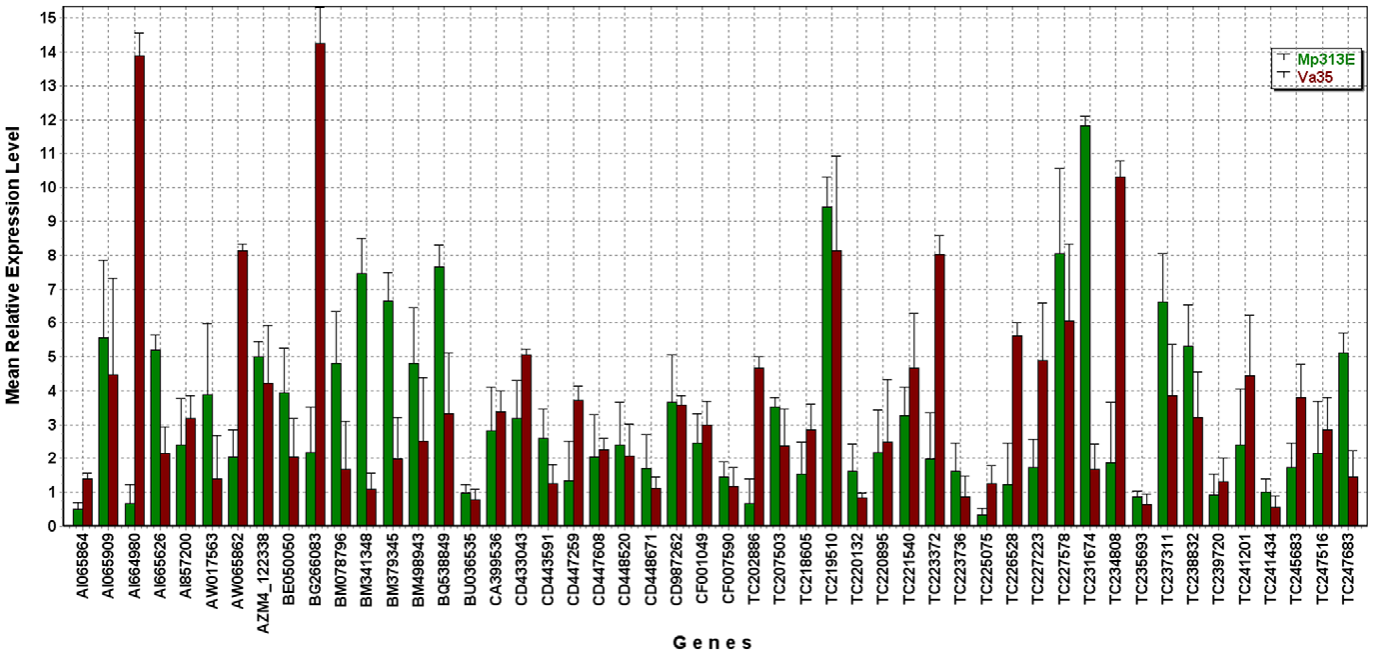
Bar-graph showing the comparison of the grand mean relative expression levels for each candidate gene in Mp313E and Va35 lines over all time points. Thirty-one of the 50 genes were found to be significantly differentially expressed (P<0.05) by a paired t-test between the Mp313E and Va35 samples. The error bars represent 95% CI values.

**Figure 3:**
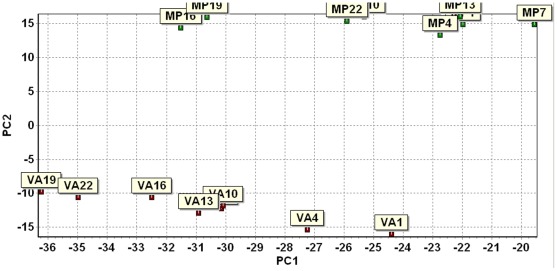
Plot showing the grouping of Mp313E and Va35 samples in the qRT-PCR study by principal component analysis. Notice the Mp313E and Va35 samples were grouped into two distinct groups. It indicated the criteria used for candidate gene selection from the microarray data were effective in reflecting host plant specific responses to the fungal infection.

**Figure 4:**
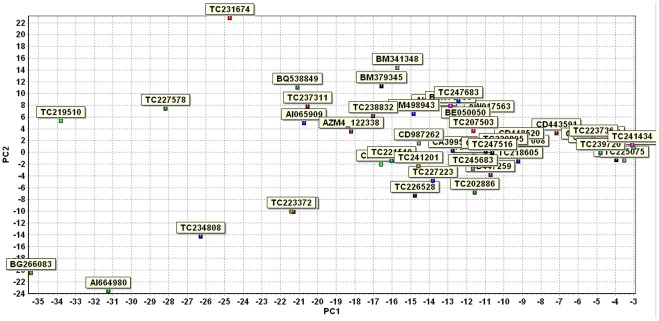
Plot of PCA analysis on the 50 candidate genes differentially expressed in Mp313E versus Va35. Genes highly expressed in Mp313E were presented in the area with positive values of y axis coordinates, whereas genes located in the area with negative values of y axis coordinates were those expressed more inVa35.

The maize microarray analysis provided a list of candidate genes possibly contributing to the host plant resistance and susceptibility. To verify and study the selected candidate genes in more details, we conducted a separate experiment for a time course quantitative study using qRT-PCR method. The resistant maize inbred line Mp313E and the susceptible maize inbred line Va35 were selected for this study. The field experimental design was a randomized complete block design with three replications for each genotype. Kernel samples were collected in the field over eight time points. The first collection was performed within three hours after the fungal inoculation on the same day (0) and the other collecting time points were at 1, 2, 3, 4, 7, 14, and 21 days after inoculation. There were three replications collected for each time point. We prepared RNA samples and performed qRT-PCR analysis to evaluate the expression levels of 50 candidate genes (Table 2) in Mp313E and Va35. The maize GAPDH gene was used for normalization. The qRT-PCR data were analyzed using the software GenEx 5. 0. 1 [20]. Figure 2 is a bar-graph showing the comparison on the grand mean expression levels for each gene between Mp313E and Va35 lines. Thirty-one of the 50 genes were found to be significantly differentially expressed (P<0.05) by a paired t-test between the Mp313E and Va35 samples (Table 3). PCA analysis on the samples showed that Mp313E and Va35 samples were classified into two distinct groups (Figure 3), which indicated that the criteria we used for candidate gene selection from the microarray data were effective in reflecting host plant specific responses to the fungal infection. Figure 4 is a plot from a PCA analysis on the 50 candidate genes to visualize group of genes differentially expressed in Mp313E versus Va35. Genes highly expressed in Mp313E were present in the area with positive y axis coordinates in this plot, whereas genes located in the area with negative y axis coordinates in this plot were those expressed more inVa35 (Figure 4).

**Table 2:** Primer sequences of candidate genes and the house keeping gene GAPDH in qRT-PCR study.

Primer ID	Primer Sequences
AI065864F	AGAATCGATCCGCCAAGTTA
AI065864R	AGGTTGCAACGCTATTGGTC
AI065909F	TACCACAGCAGAGCAACCAC
AI065909R	ATCTCCGGCTGAAGAAGACA
AI664980F	CTGACACAAAGCGACCTTCA
AI664980R	ATCCTGTTCGCTACCGTGTT
AI665626F	GGCACTGTCATCATGTTTGG
AI665626R	TATGATGCCTTCGACGATGA
AI857200F	GAAAATGACCCACCGAATTG
AI857200R	AGAGGAAGGACGCCCACTAT
AW017563F	TGTGCTCCGCTACTCAAATG
AW017563R	AACGGCCTAGATCCAATGTG
AW065862F	CGAGCGTCTTACAACAACCA
AW065862R	GGGTTCACCATGGCTAGGTA
AZM4_122338F	ATGGAACGAGGAGAAGAGCA
AZM4_122338R	TCCTGCACACACAAGAGTCC
BE050050F	CCGTGGAAATGTGGTAATCC
BE050050R	ATCCACGTCAACCATCTTCC
BG266083F	CTTTGCATCACAAAGCTCCA
BG266083R	GGTGAGGAAGAGCAAATGGT
BM078796F	TTTTCTCCACCTCGGTCTTG
BM078796R	AGCGTGAGCTCCTACGACAT
BM341348F	CTAGGAAACACCCGTCGGTA
BM341348R	CAATTGCTGCCATACAACCA
BM379345F	TTCACACACACCACACAATACC
BM379345R	CTGCAACTGTTGATCCCATC
BM498943F	CTCTGTATTGGCCCACGACT
BM498943R	AATTGTCGAGGTCGGAGATG
BQ538849F	TGATGAACAACCAAGCAAGAG
BQ538849R	ACATGGCAACGATACACGAA
BU036535F	ATCACTAATCTCACGCAACTCG
BU036535R	GCCCAAAGCTGTTGGATAGA
CA399536F	GGCTGATGCAATAAGGTGGT
CA399536R	TTGTTGCCATTCTACCCACA
CD433043F	TCTTCTTCCCCCGTACCTCT
CD433043R	AGGGCTGATGATTGTTGGAG
CD443591F	ATAGCAGCCATCCTCCATTG
CD443591R	GGGAAGAACATCCCCTTGAT
CD447259F	TCCTTGGACTTTCTGCGAGT
CD447259R	CATCACGAATACACCGTTGG
CD447608F	AAAAGGTAGCAGGGCTCACA
CD447608R	CCATTCAGCCGGTTATTTGT
CD448520F	GTGGGGCGATATTACTGCAT
CD448520R	GGGTTTCAACTGCCATTCC
CD448671F	GGATTGTCTTCCATGCAACC
CD448671R	GAAATTGCTGGGGGTAGTCA
CD987262F	ATGCGCGTACTTGCCTAAAC
CD987262R	TCAGGTACAACTCGCCCTTC
CF001049F	CTAAAAGAGGGCACCACCAA
CF001049R	TTGGCACCCTATTACAACTGC
CF007590F	GACGACGCCAGTATGTGATG
CF007590R	TAAACGAAGCTAGCGCACAA
MZ_GAPDHF	CGACTTACTTGGTGACAGCAG
MZ_GAPDHR	CGCCATCCACATTTATTCTCG
TC202886F	GAGCTTGGTGCTGGAATAGG
TC202886R	TCGCTTGAGCCTCTCTGAAT
TC207503F	AAACGCCATTGCACATTACA
TC207503R	TCTTGAAGGATCGTGTGCTG
TC218605F	AGGTTTTCGACAGCAGCAAT
TC218605R	GCCTCTTCACCAGCTAATGC
TC219510F	CTCCCTAGCCAACACACACA
TC219510R	GTCCCGGTGTAACAAACGAG
TC220132F	GTGGTCTTGACTGGGGTGTT
TC220132R	TCACAAAGCCAAAGCCTCTT
TC220895F	CAGCGAGATCAACAAGAGCA
TC220895R	GGCCCGTAGTTGTAGTTCCA
TC221540F	CAGCTGTGGCAGGACTACAA
TC221540R	TCATACCAAACGCATTGAGC
TC223372F	AGGATCTGGGGATGGATTTC
TC223372R	CATGCATGGTCGTGTTTTGT
TC223736F	AACGGTCAGAATTGGAGTGC
TC223736R	GACGACGCAACAGATCTCAA
TC225075F	CTGCTGATCGAGACATTGGA
TC225075R	AAGACATGCAACCAACACCA
TC226528F	TGTTCGTCCTCTGCTTGTTG
TC226528R	TAATGGGTGGAAGGAATGGA
TC227223F	CAGCCAAGATGTTTGCATTG
TC227223R	ATCCATGGGTTCATGGTAGC
TC227578F	AGCGTGAGCTCCTACGACAT
TC227578R	CAACCCACGCTAGTGCTACA
TC231674F	GGGCTTCTTGTTGTGCTCTC
TC231674R	TTAAAGCGCTGCCTTATTCC
TC234808F	AATGTCGACCTTGGAACTGC
TC234808R	CTGCAGGGGCTTCTTTACTG
TC235693F	GCAAGCTTCTGGTCATCCTC
TC235693R	TTCTCTGCAACAATGCCAAC
TC237311F	TGAGGATCATGGAGGAGGAC
TC237311R	CCACATTCACGGGCTTATCT
TC238832F	AGACATGGGATACCGAGACG
TC238832R	AGCTCCATCAGCTCCTTGAA
TC239720F	GATTCCTGATCCGAAGGACA
TC239720R	TTCCAAGGTCCCTTGTATGC
TC241201F	TCTTTCGACTGGTGATGCTG
TC241201R	CCCCACTGCATGTAGGACTT
TC241434F	ACAACCTCACCTTTGCAACC
TC241434R	GCTGCTATGTACGCCATCAA
TC245683F	CGGAATGGTACTCCTGGTTG
TC245683R	TGGGAGTCTCACACTCACGAT
TC247516F	AGGGCACCATGAGAAATCTG
TC247516F	AGGGCACCATGAGAAATCTG
TC247516R	GAATGCTGGTCCTGTTGGAT
TC247516R	GAATGCTGGTCCTGTTGGAT
TC247683F	ATGATGGGAGGCTGACTTTG
TC247683R	TCTCAGCGAAATTCATCGTG

**Table 3:** Genes differentially expressed in the resistant maize inbred line Mp313E and the susceptible maize inbred line Va35 verified by qRT-PCR method.

Host Plant	ID	Chr	Bin	Function
**Mp313E**	BM078796***	Chr 1	1.03	Heat Shock Protein 26 (HSP26)
	TC238832 **	Chr 2	2.06	Lecithin cholesterol acyltransferase (LCAT)
	BM498943 ***	Chr 3	3.08	Ethylene Responsive Protein (ETHRP)
	BQ538849 **	Chr 4	4.00	C2H2-type Family Protein
	BM379345 ***	Chr 4	4.01	Metallothionein-Like Protein (MTLP)
	BM341348***	Chr 4	4.01	Zein
	BE050050 *	Chr 4	4.05	In Chr4 QTL
	TC223736 *	Chr 4	4.10	Heat Shock Protein 90 (HSP90)
	TC241434 *	Chr 5	5.03	
	TC231674 ***	Chr 5	5.05	NPCs-NUP85, RNA Transport
	CD443591 *	Chr 6	6.04	
	AI665626***	Chr 6	6.05	Zein
	TC237311*	Chr 6	6.07	Heat Shock Protein (HSP 101)
	TC207503 *	Chr 8	8.03	Prenylated Rab Acceptor (PRA1) Family Protein
	TC247683 ***	Chr 8	8.05	
	AW017563* ^a^	NA	NA	
**Va35**	AI664980 ***	Chr 1	1.06	Glycine Rich RNA Binding Protein2(GRBP2), in Chr1 QTL
	TC218605 *	Chr 1	1.09	Phytochrome A (PHYA)
	TC241201*	Chr 2	2.07	
	TC202886 ***	Chr 2	2.08	
	TC226528 **	Chr 3	3.05	Uracil Permease (UPS)
	AW065862***	Chr 4	4.01	Zein
	CD433043 *	Chr 4	4.01	Zein
	CD447259*	Chr 4	4.01	Zein
	AI065864**	Chr 5	5.05	Exonuclease-Endonuclease-Phosphatase (EEP)
	TC225075*	Chr 5	5.07	Choline Transport
	TC245683 **	Chr 6	6.02	
	TC227223 *	Chr 6	6.05	Zein
	TC223372 ***	Chr 7	7.02	Cinnamoyl CoA Reductase (CNCR 2)
	TC234808 ***	Chr 8	8.01	Ribosomal Protein L30 (RPL30)
	BG266083 ***	Chr 9	9.05	HSP18a

* p value <0.05, **p value <0.001, ***p value <0.0001. The significance levels were determined by a paired t-test for the qRT-PCR data. ^a^ AW017563 chromosome and bin information is not available.

### Genes Highly Expressed in Resistant Maize Inbred Line Mp313E

**Figure 5:**
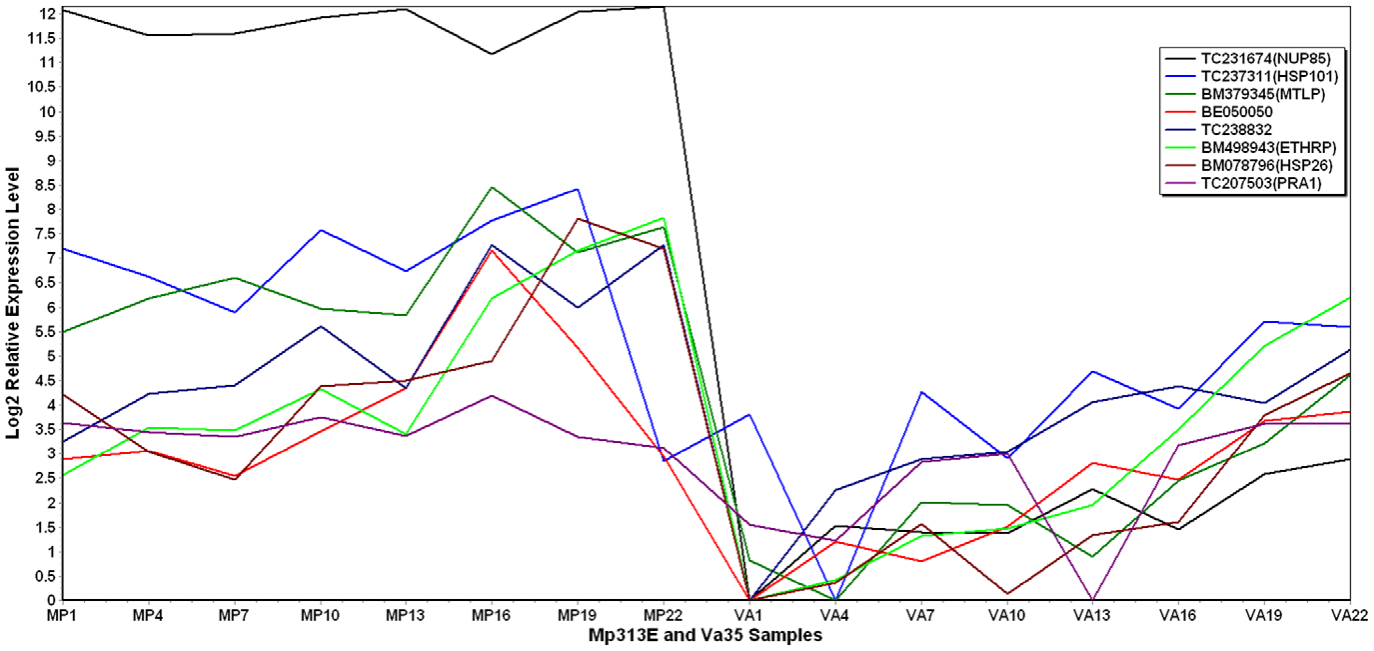
2D plot showing gene expression levels over a period of 21 days after the fungal inoculation. These genes are highly expressed in Mp313E than in Va35.

Sixteen of the 50 candidate genes showed significantly higher expression levels in the resistant maize inbred line Mp313E than in the susceptible line Va35 (Table 3). The gene functions were determined by searching the Genbank and the Maize GDB. Figure 5 is a 2-D plot showing quantitative comparison for some of the significant genes over a period of 21 days after inoculation with the fungus in Mp313E and Va35. TC231674 is the highest expressed found in Mp313E samples. TC231674 encodes a NUP85-like gene that is a part of a sub-complex of the nuclear pore complexes (NPCs) embedded in the nuclear envelope. The function of NPCs is for the transport of RNA and other macromolecules from nucleus to cytoplasm. TC237311 and BM379345 are second highest in expression. TC237311 encodes the heat shock protein HSP101. This protein acts as a molecular chaperone to disaggregate mis-folded proteins. BM379345 encodes a metallothionein like protein (MTLP) that is involved in the binding and detoxification of heavy metal ions. BE050050 and TC238832 comprise the next level in expression. BE050050 has no annotation available. TC238832 encodes a lecithin cholesterol acyltransferase (LCAT)-like gene. Other highly expressed genes include BM498943, BM078796, and TC207503. BM498943 encodes ethylene responsive protein (ETHRP) that belongs to the universal stress protein family. BM078796 encodes small heat shock protein HSP26. TC207503 encodes a prenylated rab acceptor (PRA1) family protein. Prenylated Rab PRA1 proteins are small transmembrane proteins that regulate vesicle trafficking.

### Genes Significantly Expressed More in the Susceptible Maize Inbred Line Va35

**Figure 6:**
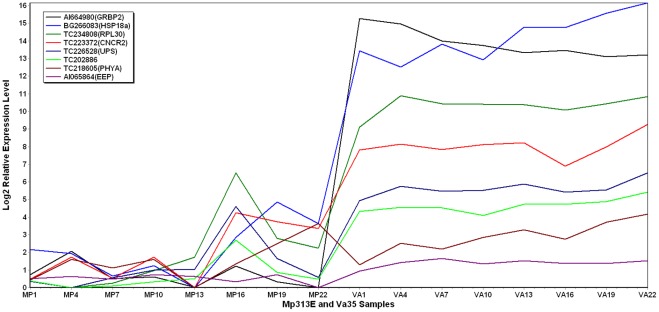
2D plot showing gene expression levels over a period of 21 days after the fungal inoculation. These genes are highly expressed in Va35 than in Mp313E.

Fifteen genes were found to be significantly expressed more in the susceptible line Va35 than in the resistant line Mp313E (Table 3). The highest expressed genes found in Va35 were AI664980 and BG266083 (Figure 6). AI664980 belongs to the glycine-rich RNA binding protein family (GRBP2) that has RNA binding domains (RBD) commonly found in proteins involved in post-transcriptional gene expression processes. BG266083 encodes alpha-crystallin-type of stress-induced small heat shock proteins (HSP18a). TC234808 and TC223372 were also highly expressed in Va35. TC234808 is in the ribosomal protein L30 family (RPL30). The ribosomal protein L30 has pre-mRNA splicing regulatory activity to its own transcript and plays a key role in the assembling of the ribosomal subunits. TC223372 is a cinnamoyl-CoA reductase (CNCR2) that catalyses the lignin pathway. Other highly expressed genes found in Va35 included TC218605, TC226528, and AI065864. TC218605 encodes phytochrome A (PHYA). TC226528 encodes a ureide permease (UPS) that transport and recycle organic nitrogen for nucleotide synthesis. AI065864 belongs to the exonuclease-endonuclease-phosphatase (EEP) domain superfamily (Figure 6). There is no annotation for TC202886 in the database.

### Mapping of the Highly Expressed Genes to the QTL Maps

**Figure 7:**
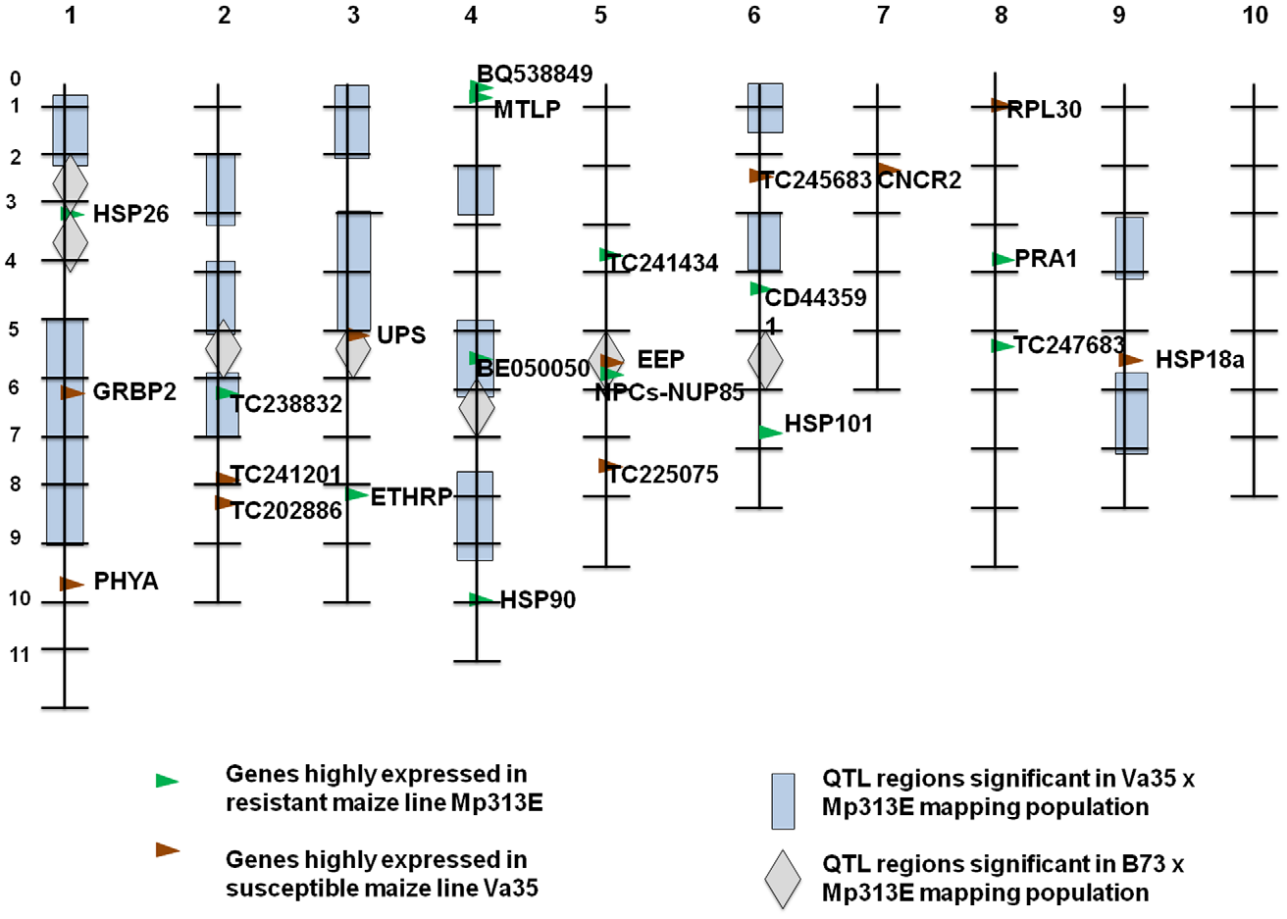
Chromosome bin map showing the positions of the significant genes and the previously identified QTL regions. Eight genes were mapped within or close to the seven most significant QTL regions. The top highly expressed gene in Va35, AI664980 (Chr1, bin1.06), is located in the most significant chromosome 1 QTL region (Chr 1, bin 1.5–1.9) identified from the Va35 x Mp313E population. The top highly expressed gene in Mp313E, TC231674 (Chr 5, bin 5.05), is mapped to the chromosome 5 QTL region (Chr 5, bin 5.05) identified from B73 x Mp313E population.

Figure 7 is a maize chromosome bin map showing the identified QTL regions from previous studies on two QTL mapping populations [14, 15, and Willcox et al. 2000, unpublished data]. We mapped the highly expressed candidate genes and the previously identified QTLs on the chromosome bin map to compare their relative chromosomal positions. Seven genes were mapped within and one gene close to the most significant QTL regions identified from the two mapping populations (Figure 7). The top highly expressed gene in Va35, AI664980 (Chr1, bin1.06), is located in the most significant chromosome 1 QTL region (Chr 1, bin 1.5–1.9) identified from the Va35 x Mp313E population. BG266083 (Chr 9, bin 9.05) is located close to the chromosome 9 QTL region (Chr 9, bin 9.06–9.07). The top highly expressed gene in Mp313E, TC231674 (Chr 5, bin 5.05), is mapped to the chromosome 5 QTL region (Chr 5, bin 5.05) identified in B73 x Mp313E population. BE050050 (Chr 4, bin 4.05) has no known function, but it is located within the chromosome 4 QTL region (Chr 4, 4.05–4.06) identified from both of the mapping populations. TC238832 (Chr 2, bin 2.06) is within a chromosome 2 QTL region (Chr 2, 2.06–2.07) from the Va35 x Mp313E population and close to a QTL region (Chr 2, bin 2.05) in the B73 x Mp313E population. BM078796 (HSP26) (Chr1, bin 1.03) is located in the chromosome 1 QTL region (Chr1, bin 1.03) in B73 x Mp313E population. These findings will be important in the identification of appropriate DNA markers for breeding of *Aspergillus flavus* and aflatoxin resistance maize lines.

## Discussion

*Aspergillus flavus* infection is a major concern for maize producers. Constant efforts are being made by plant breeders to develop resistant genotypes. Identification of genes having larger effects on the resistance or susceptibility is important to facilitate molecular marker-assisted breeding of resistant maize lines. Here, we evaluated different statistical tools for microarray data analysis, combined different methods to select the potentially relevant genes, and then conducted further quantitative expression analysis to take a closer look at the highly expressed host plant specific genes. Despite the complexity of the genotypic and environmental effects, the resistant and susceptible maize inbred lines exhibited distinguishable gene expression patterns in response to *Aspergillus flavus* colonization. These findings indicate that there are general maize host plant- *Aspergillus flavus* recognition and interaction processes that underlie resistance and susceptibility.

The susceptible maize inbred line Va35 showed hypersensitivity in response to *Aspergillus flavus* infection. Two highly expressed genes in Va35 are known to play roles in the plant responses toward various stress and pathogens. AI664980 encodes a glycine-rich RNA binding protein (GRBP2). A number of plant GRBPs were originally characterized as a result of cloning stress responsive genes. GRBPs were found to be associated with a variety of biotic and abiotic stresses including hormone, temperature, wounding, and pathogen infection [21], [22]. It was demonstrated that a tobacco GRBP gene was differentially expressed during HR reaction and it was also inducible by exogenous salicylic acid, suggesting that it plays roles in plant defense signal transduction and HR reaction [21]. GRBPs function through binding to RNA molecules and interfering with the post-transcriptional modifications. It has also been shown that GRBPs can interact with multiple proteins and RNA molecules, including its own mRNA, which is a feed-back mechanism for regulatory proteins [23]. TC234808 encodes a protein in the ribosomal protein L30 (RPL30) family. A RPL30 protein binds to its own transcript to inhibit the splicing and the translation of its own mRNA when it is expressed in excess. RPL30 proteins are important for governing the large and small ribosomal subunits assembling [24]. Both AI664980 and TC234808 genes have RNA binding domains and are likely involved in the post-transcriptional regulation of the genes in plant defense systems. BG266083 encodes alpha-crystallin-type of stress-induced small heat shock proteins (sHSPs) namely HSP18a. The sHSPs proteins are ubiquitous stress proteins that act as chaperones to prevent protein aggregations [25]. The roles of other highly expressed genes found in Va35 in response to *Aspergillus flavus* infection are not clear. It may be worth mentioning that TC226528 encodes a ureide permease (UPS) that transports and recycles organic nitrogen for nucleotide synthesis, indicating elevated activities in the metabolism of RNA molecules in Va35. AI065864 belongs to an exonuclease-endonuclease-phosphatase (EEP) domain superfamily. Enzymes in this large superfamily have the catalytic domain for cleaving phosphodiester bonds in nucleic acids. TC223372 encodes a cinnamoyl-CoA reductase (CNCR2) that catalyses the first step of the lignin pathway. The high level of TC223372 gene expression suggests that cell wall lignifications are probably involved in the infected Va35 plants.

The observed hypersensitive expression of stress responsive genes in Va35 appeared to be associated with its susceptibility to *Aspergillus flavus*. The hypersensitive response in Va35 likely causes cell death, and as a result, *Aspergillus flavus* infects and colonizes the host plant. In contrast, only very low level of AI664980 and TC234808 mRNAs were detected in the resistant maize inbred line Mp313E, which likely indicates that an insensitivity mechanism of the resistance is involved in Mp313E in response to the *Aspergillus flavus* infection. This hypothesis can explain some evidence found in the previous QTL studies. For example, AI664980 (Chr 1, bin 1.06) is located within the chromosome 1 QTL region (Chr 1, bins 1.05–1.09) identified in the Va35 x Mp313E population (Figure 7). This QTL was the most significant one and was identified from each of the planting locations and over all the three years (14, and Willcox et al. 2000 unpublished data). However, the resistance source of this QTL appeared to be associated with the susceptible Va35 genotype. Because this QTL had a big span and there were only a few polymorphic DNA markers available at the time, it is likely that there were crossovers that were not detected in that region, and the resistance was actually from the insensitive Mp313E allele. Similarly, it was possible for the reason of lacking suitable DNA markers that BG266083 (HSP18a) (Chr 9, bin 9.05) was mapped close to, but not within the chromosome 9 QTL (Chr 9, bin 9.06–9.07) region, and TC234808 (RPL30) (Chr 8, bin 8.01) was undetected in the previous QTL study. More information will come from an ongoing cloning and sequencing project for different alleles of these genes.

In addition to its apparent lacking of a sensitive form of the AI664980 allele, the resistant maize inbred line Mp313E appears to possess other resistance mechanisms as well. Evidence from our quantitative expression analysis showed that genes encoding RNA transport regulators, molecular chaperones, and detoxification proteins were highly expressed in Mp313E. The highest expressed gene TC231674 (Chr 5, bin 5.05) is homologous to the human nucleoporin NUP85 which is a component of the nuclear pore complexes (NPCs) in nuclear envelope. Nucleoporins are conserved from yeast to human. NPCs are involved in the transport of RNA and other macromolecules which is a fundamental regulatory process for plant defense system [26]. Recent studies have strongly suggested that components of NPCs regulate the transport of R proteins [26], [27]. A partial loss-of-function MOS7/NUP88 mutant gene in *Arabidopsis* was found to be associated with the suppression of the R protein *snc1* mediated resistance [27]. In our study, we found that the NUP85-like nucleoporin gene was highly expressed in the resistant line Mp313E. In contrast, only low expression level of this gene was found in the susceptible line Va35. This indicates that the NPCs-related regulatory activities are involved in the maize host resistance associated with Mp313E. The highly expressed molecular chaperones in Mp313E included heat shock proteins HSP26, HSP90, and HSP 101. In addition, BM498943 encodes ethylene responsive protein (ETHRP) that belongs to the universal stress protein family. The universal stress proteins confer stress endurance and increase cell survival rate in general. TC207503 encodes a prenylated rab acceptor (PRA1) protein. Prenylated Rab PRA1 proteins are small transmembrane proteins that are involved in intracellular vesicle trafficking and the secretory pathways in cells. BM379345 encodes a metallothionein like protein (MTLP) that is involved in the binding and detoxification heavy metal ions. It was found that increased Aflatoxin production was associated with high levels of certain trace metal elements [28]. Evidence supporting the multiple resistance mechanisms present in Mp313E was also obtained from the previously conducted QTL studies in the B73 x Mp313E and Va35 x Mp313E populations. Two significant resistance QTLs were found to be associated with Mp313E genotype [14, 15, and Willcox et al. 2000, unpublished data], indicating that multiple defense systems are involved in Mp313E. Interestingly, the susceptible maize inbred line B73 appeared to have a different allele of AI664980 from Va35 (data not show), indicating that there are also multiple mechanisms underlying maize host susceptibility in response to *Aspergillus flavus* infection.

The resistance of maize to *Aspergillus flavus* infection and aflatoxin accumulation has been investigated from various aspects [29], [30]. However, this is the first comprehensive quantitative expression analysis to characterize genes associated with maize host plant hypersensitive responses and susceptibility in Va35, to identify genes that are associated with resistance in Mp313E, and to map quantitatively verified genes to previously identified major QTL regions. Our findings suggest that a combination of microarray analysis, qRT- PCR analysis, and QTL mapping can provide an efficient strategy to discover genes associated with *Aspergillus flavus* infection and aflatoxin accumulation.

## Materials and Methods

### Plant Materials and Experimental Designs

Maize inbred lines Mp313E, Mp04∶86, Va35, and B73 were maintained by the United States Department of Agriculture, Agricultural Research Service, Corn Host Plant Resistance Research Unit (USDA-ARS-CHPRRU) at Mississippi State University. Mp313E and Mp04∶86 are maize inbred lines showing resistance to *Aspergillus flavus* infection and aflatoxin accumulation. Va35 and B73 are elite maize germplasm but are susceptible to *Aspergillus flavus*. Mp04∶86 is a recombinant inbred line derived from a cross of maize lines Va35 and Mp715. Both Mp715 and Mp313E are used primarily as resources of resistance for maize breeding projects [31], [32]. For the microarray experiment, the field experimental design was a randomized complete block with split plot and three replications for each genotype. All maize lines were planted at the R. R. Foil Plant Science Farm at Mississippi State University. The four genotypes were planted in the main plots. The two treatments (inoculated and uninoculated) were applied to the corresponding subplots. All primary ears were self-pollinated. The inoculation was performed 14 days after self-pollination using the fungus *Aspergillus flavus* strain NRRL 3357 (ATCC # 200026; SRRC 167), a strain known to produce high levels of aflatoxin in corn grain [33]. The procedure of fungal culture preparation and the fungal inoculation with side-needle teqhnique was as described previously [30], [34]. Four days after inoculation, which was 18 days after self-pollination, kernels from inoculated and uninoculated primary ears were collected for RNA preparation. The experimental procedure for microarray hybridization and data acquisition followed Kelley et al [30]. All remaining primary ears from each plot were harvested at maturity and processed as previously described by Windham and Williams [35] for aflatoxin accumulation analysis.

The field experimental design for qRT-PCR analysis was a randomized complete block with three replicates for each genotype. The resistant Mp313E and the susceptible Va35 were used. Kernel samples were collected in the field over eight time points. The first collection was performed within three hours after the fungal inoculation (0 days after inoculation). The other collecting time points were at 1, 2, 3, 4, 7, 14, and 21 days after inoculation. Kernels were flash frozen in liquid nitrogen, ground into powder, and stored at –80°C for further analysis.

### Maize Oligonucleotide Microarray Hybridization and Data Acquisition

The maize oligonucleotide arrays from the NSF Maize Oligonucleotide Array Project were used. Each set of the maize arrays contains two slides, MO-A-1-9 and MO-B-1-9, with 57,452 maize gene oligonucleotide probes altogether [36]. Each slide was hybridized with the inoculated and uninoculated samples of the same genotype from the same main plot. Three sets of slides for six samples were used for Mp313E and B73, respectively. Two sets of slides for four samples were used for Mp 04∶86 and Va35, respectively. An additional set of slide was used as a dye swap for each genotype. The experiment followed the procedure as described previously [30]. All recommendations of the minimum requirements for a microarray experiment (MIAME) checklist [37] were observed, and the microarray data have been deposited with the European Molecular Biology Laboratory (EMBL) – the European Bioinformatics Institute (EBI) (E-MTAB-766).

### Microarray Data Analysis

All data were first analyzed with SAS Version 9.1.3 (SAS, Cary, NC) [18]. A more detailed description of the method can be found in Kelley et al [30]. Briefly, the median value for the intensity of each spot was log transformed and analyzed by analysis of variance appropriate as a split plot design. The main unit was genotype and the subunit was inoculation treatment. Dye was treated as a fixed effect in the model to account for differences in dyes. Estimates of expression ratios (I/U) for each gene of each genotype were obtained. F-test for Genotype x Inoculation Treatment interaction was used to address the null hypothesis and obtain the significance levels for the expressed genes. Here is an example for an equation of the null hypothesis between a resistant maize line (Mp313E) and a susceptible maize line (Va35). H0: Log (Mp313E-I) - Log (Mp313E-U)  =  Log (Va35-I) - Log (Va35-U), where I and U refer to inoculated and uninoculated, respectively. Finally, the log2 values of the expression ratios (I/U) for expressed genes in all genotypes were analyzed by principal component analysis (PCA) and plotted using R 2. 12. 1 [38].

The microarray data were also analyzed using the R package of the Linear Models for Microarray Data (LIMMA) [19]. For the preprocessing step, data were normalized by the within array print group loess method. Log ratio (M) and log intensity (A) were calculated. Highly significant genes (P<0.01) were found and ranked via the limFit and topTable functions.

### Quantitative Real Time RT-PCR

To validate the expression levels of candidate genes obtained by microarray analysis, qRT-PCR [39] was conducted using the Roche LightCycler 480 instrument (Roche Applied Science). ThermoScript RT-PCR system (Invitrogen, #11146-024) was used to prepare cDNA samples. LightCycler 480 SYBR Green I Master kit (Roche Applied Science, #04 707 516 001) was used for the qRT-PCR reactions. The qRT-PCR program was as the following: 1) 1 cycle of 95°C for 5 min; 2) 45 cycles of 95°C for 10 sec, 60°C for 15 sec, 72°C for 15 sec; 3) 1 cycle of 95°C for 5 sec, 65°C for 1 min, 97°C at continuous; 4) 1 cycle of 40°C for 10 sec. The primer sequences for qRT-PCR are listed in Table2. Data were analyzed and plotted by using GenEx 5.0.1 [20]. The PCR efficiency correction and reference gene normalization followed the software instructions. Two housekeeping genes, *Zea mays* glyceraldehyde-3-phosphate dehydrogenase (GAPDH) and *Zea mays* RNA polymerase II largest subunit (RPII), were tested in this qRT-PCR study. The geNorm function in GenEx was used to determine which housekeeping gene had least variations among all the samples. The gene has the smallest M-value was used as a reference gene. For the qRT-PCR statistical analysis, the paired t-test (2-tail) was used to test the two means of the expression levels of each gene between Mp313E and Va35 samples. The null hypothesis is H0: no difference between the expression level means in Mp313E and in Va35 for the tested gene.

### Maize Chromosome Bin Location Comparison of Candidate Genes and QTLs

Information of QTLs used in this study was obtained from two mapping populations studied previously. The Mp313E xVa35 population includes 216 F3 families and was evaluated for three years. A total of 15 QTL regions, located on chromosomes 1, 2, 3, 4, 6, and 9, have been identified (Figure7). Four QTLs on chromosome 1, 4 (2), and 9 were above a significance level of 23.58 in likelihood ratio [14, and Willcox et al. 2000, unpublished data]. Two regions (chromosome 1, bin 1.08–1.09) and chromosome 4 (bin 4.04–4.08) were identified in all three years. The chromosome 1 (bin 1.08–1.09) QTL was associated with the Va35 genotype of DNA marker. The chromosome 4 (bin 4.04–4.08) QTL was associated with the Mp313E genotype of DNA marker. The Mp313E x B73 population contains 210 F2∶3 families and was also evaluated for three years [15]. A total of seven QTL regions were identified. They are located on chromosome 1, 2, 3, 4, 5, and 6 (Figure 7). The two QTLs on chromosome 2 and 4 were most significant, followed by the two QTLs on chromosomes 3 and 5 in this population.

The genetic map of each maize chromosome is divided into 100 segments, namely bins, which are marked by the Core Bin Markers [40]. A bin comprises all loci between the two Core Bin Markers. The information of the mapped loci in each bin is available in the Maize Genetics and Genome database (the Maize GDB). We therefore placed QTLs from different populations on one chromosome bin map for display purpose (Figure 7). Different legends were used in Figure 7 to distinguish the population origins of the QTLs. The physical mapping locations of the Core Bin Markers are identified also in the Maize GDB. The chromosomal bin locations of the highly expressed genes were determined by searching for gene sequence positions in the Maize GDB and then comparing them with the physical positions of the Core Bin Markers used for QTL analysis. Figure 7 shows a combined maize chromosome bin map for the purpose of display and comparison of results from multiple experiments.
